# Novel tumor suppressor role of miRNA-876 in cholangiocarcinoma

**DOI:** 10.1038/s41389-019-0153-z

**Published:** 2019-08-13

**Authors:** Sarah Ursu, Shahana Majid, Caroline Garger, David de Semir, Vladimir Bezrookove, Pierre-Yves Desprez, Sean McAllister, Liliana Soroceanu, Mehdi Nosrati, Kidist Yimam, Assad Hassoun, Robert Osorio, Mohammed Kashani-Sabet, Altaf A. Dar

**Affiliations:** 1 California Pacific Medical Center Research Institute, 475 Brannan St, Suite 130, San Francisco, CA 94107 USA; 2 Department of Urology, Veterans Affairs Medical Center and University of California San Francisco, San Francisco, CA 94121 USA

**Keywords:** Oncogenes, Biliary tract cancer

## Abstract

Cholangiocarcinoma (CCA) is a rare, highly invasive malignancy, and its incidence is increasing globally. MicroRNAs (miRNAs) mediate a wide array of cellular and biological processes and are dysregulated in various tumors. The functional and biological roles of miRNAs in CCA have not been fully elucidated. In this study, we show that miR-876 expression levels and copy number are significantly attenuated in the TCGA cohort of CCA tissue samples. TCGA expression data was consistent with the observed substantial decrease in miR-876 expression in patient samples and CCA cell lines. In-silico algorithm databases revealed BCL-XL as a potential target of miR-876. We observed miR-876 expression to be downregulated, whereas, BCL-XL upregulated in CCA cell lines. BCL-XL was identified as a direct functional target of miR-876 in CCA. miR-876-mediated reduction of BCL-XL regulated cell survival, induced apoptosis and caspase 3/7 expression in CCA. BCL-XL overexpression reversed the miR-876 mediated effect on CCA cell growth and apoptosis. Stable overexpression of miR-876 produced potent tumor suppressor activity and in vivo tumor cell growth reduction. Overexpression of miR-876 in a patient-derived xenograft (PDX) cell line significantly suppressed BCL-XL expression and spheroid formation with a concomitant induction of caspase 3/7 activity and apoptosis. This study demonstrates a novel tumor suppressor role for miR-876 in CCA, identifies BCL-XL as an actionable target, and suggests a potential therapeutic role for miR-876 in CCA.

## Introduction

Cholangiocarcinoma (CCA) is highly invasive epithelial cancer of the biliary tree with a high mortality rate^1^. Due to dearth of clinical indications, CCA in not easily diagnosed at early stages. CCA patients comprise 10–20% of hepatobiliary cancers^2^, however, incidences and mortality of CCA are increasing rapidly worldwide^1,3^. Currently, there is no clinical treatment for CCA, with resection representing the only possibility for long-term survival^4^. CCA patients have a dismal 5 year survival rate of 10%, hence there is an urgent need to identify novel therapies for CCA^5^.

MicroRNAs (miRNAs) are small endogenous single strand RNAs of 18–22 nucleotides that modulate gene expression of its targets either by degradation and/or repression of translation^6,7^. miRNAs mediate key roles in different biological processes, such as cell cycle, proliferation, apoptosis, migration, and invasion^8,9^. miRNAs are either upregulated or downregulated in different tumors, including CCA, and have been shown to act as oncogenes or tumor suppressors^4,8,10^. However, to date, the functional roles of miRNAs in CCA are not fully elucidated. Here, we report a biological role for miR-876 in CCA progression, identify BCL-XL as its functional target in established cell lines and in a patient-derived xenograft culture, and suggest a potential therapeutic role.

## Results

### miR-876 is suppressed in CCA tissues and cell lines

**Fig. 1:**
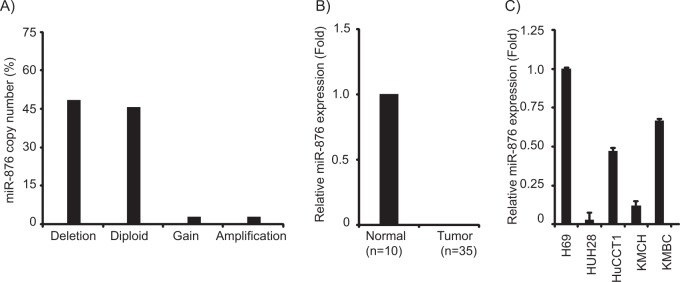
miR-876 is suppressed in cholangiocarcinma (CCA). **a** Copy number analysis of CCA samples from TCGA database. **b** Relative miR-876 expression levels of CCA samples from TCGA database. **c** Relative miR-876 expression levels in a normal human cholangiocyte line (H69) and cholangiocarcinoma cell lines

Analysis of The Cancer Genome Atlas (TCGA) database of CCA samples revealed a substantial copy number loss of miR-876 (17/35, 48.5%) (Fig. 1a). Further, analysis of these samples identified a significant suppression of miR-876 expression in tumor samples as compared with samples from normal individuals (Fig. 1b). miR-876 expression was substantially downregulated in CCA cell lines as compared with a normal human cholangiocyte (Fig. 1c). Expression of miR-876 was analyzed in nine patient tumor samples and was undetectable by miRNA qRT-PCR analysis, indicating a low expression level. This analysis demonstrated a possible tumor suppressor function for miR-876 in CCA.

### miR-876 targets BCL-XL

**Fig. 2:**
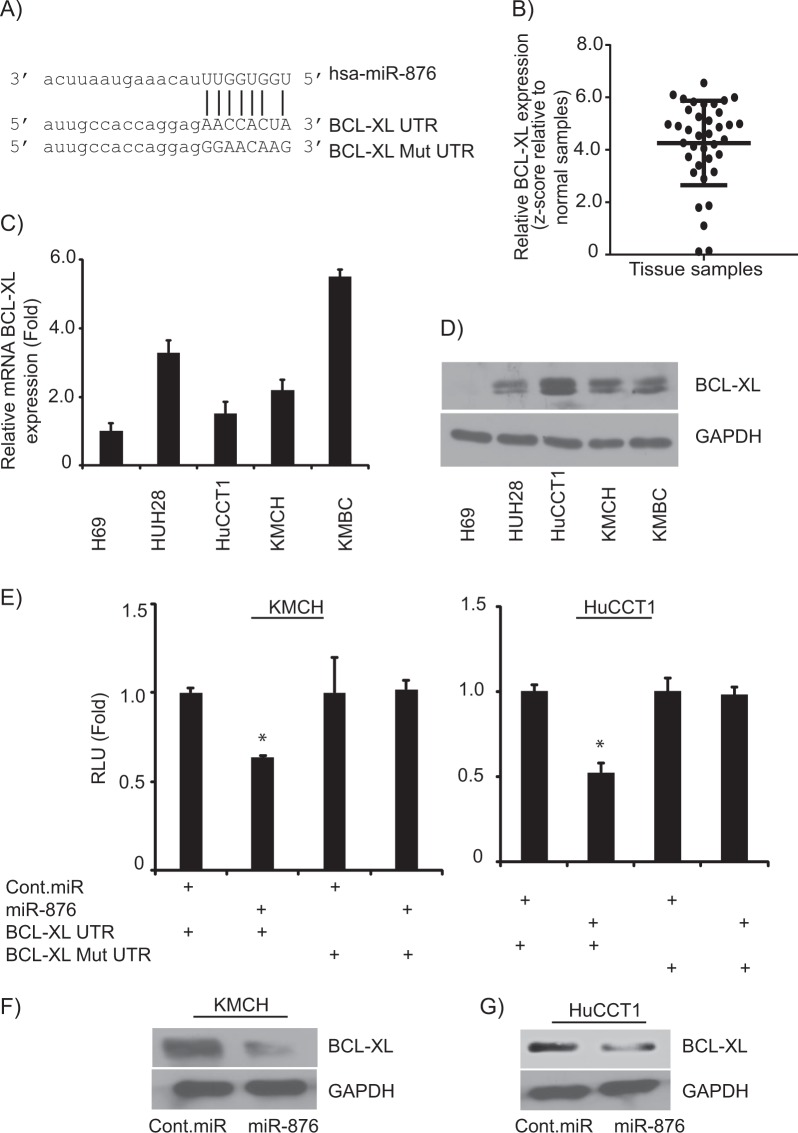
BCL-XL is a target of miR-876 action. **a** The seed sequence of miR-876 is complementary to the 3′UTR of BCL-XL. **b** BCL-XL expression levels in CCA samples relative to normal samples. **c**, **d** BCL-XL mRNA and protein expression levels in a normal human cholangiocyte line and CCA cell lines. **e** Luciferase activity after miR-876 overexpression in KMCH and HuCCT1 cell lines. **f**, **g** BCL-XL protein expression following miR-876 overexpression in KMCH and HuCCT1 cell lines. *p < 0.05

To investigate potential targets of miR-876, in-silico algorithm databases (targetscan.com, microrna.org) were employed to predict its putative target(s) of action. This analysis identified BCL-XL as one potential target, due to the complementarity of the 3′UTR of BCL-XL to the seed sequence of miR-876 (Fig. 2a). We analyzed BCL-XL expression in CCA samples in TCGA database and found it to be overexpressed in tumor samples when compared with normal samples (Fig. 2b). Expression of BCL-XL at mRNA (Fig. 2c) and protein levels (Fig. 2d) were elevated in CCA cell lines, when compared with normal cholangiocyte line. These results affirm that miR-876 expression is downregulated in CCA cell lines, whereas, BCL-XL expression level is upregulated in CCA cells.

To confirm BCL-XL as a direct and functional target of miR-876, BCL-XL 3′UTR having the target sequence complementary to miR-876 was cloned in a pmirGLO dual-luciferase vector. Transfection of miR-876 along with the BCL-XL 3′UTR construct into KMCH and HuCCT1 CCA cell lines resulted in a significant downregulation in luciferase expression as compared with negative control pre-miRNA (cont.miR) (Fig. 2e). Transfection of mutated BCL-XL 3′UTR along with miR-876 did not alter luciferase reporter activity (Fig. 2e). A significant suppression in BCL-XL expression was observed at the protein level in CCA cell lines following miR-876 overexpression (Fig. 2f, g), without having any effect on expression of BCL-XL at mRNA level (Supplementary Fig. 1). We also analyzed the role of miR-876 on BCL2 and MCL-1 expression, other family members of BCL-XL. miR-876 overexpression had no effect on BCL2 and MCL-1 protein expression levels in KMCH and HuCCT1 cell lines (Supplementary Fig. 2A, B). These results indicate that miR-876 specifically targets BCL-XL.

### miR-876 overexpression regulates cell survival

**Fig. 3:**
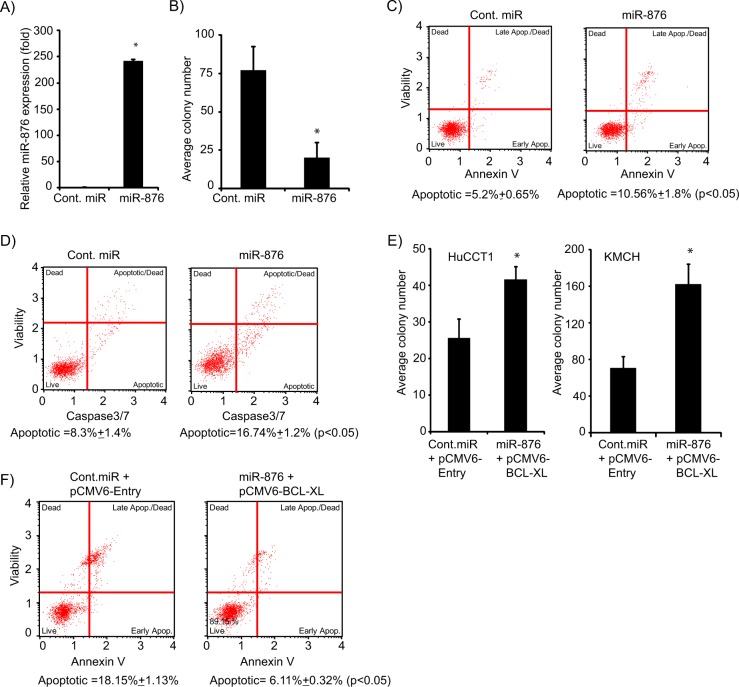
miR-876 overexpression regulates cellular proliferation and induces apoptosis. **a** miR-876 overexpression in KMCH cells. **b** Average colony number of KMCH cells following miR-876 overexpression. **c** miR-876 overexpression induces apoptosis in KMCH cells. **d** miR-876 increases caspase 3/7 activity in KMCH cells. **e** Average colony number following miR-876 and pCMV6-BCL-XL co-overexpression in HuCCT1 and KMCH cell lines. **f** Apoptosis analysis following miR-876 and pCMV6 BCL-XL co-overexpression in KMCH cell line. *p < 0.05

To determine the functional effects of miR-876 on CCA cell lines, miR-876 was transiently overexpressed in the KMCH cell line (Fig. 3a). A substantial suppression in ability to form colonies was observed in miR-876 overexpressing cells as compared with cells expressing cont. miR (Fig. 3b). To validate the effects of miR-876 on cell proliferation, HuCCT1 cells were transfected with miR-876 (Supplementary Fig. 3A) and it substantially suppressed the colony numbers. (Supplementary Fig. 3B). These findings suggest that miR-876 regulates CCA cell proliferation and growth, possibly due to its regulation of BCL-XL expression levels.

### miR-876 overexpression induces apoptosis

Next, we determined whether miR-876 had any effect on apoptosis, given BCL-XL’s known role as an antiapoptotic gene. Overexpression of miR-876 in KMCH cells significantly increased the percentage of cells undergoing apoptosis. (Fig. 3c). Caspase 3/7 activity was significantly increased in miR-876-expressing cells in comparison to cells expressing cont.miR (Fig. 3d). To validate the effects of miR-876 overexpression on apoptosis and caspase 3/7 activity, miR-876 was overexpressed in the HuCCT1 cell line. We observed similar increases in the apoptotic population and caspase 3/7 activity. (Supplementary Fig. 3C, D). In order to further investigate the role of BCL-XL as a miR-876 target, CCA cells were cotransfected with miR-876 along with BCL-XL cDNA vector. mir-876 cotransfection with BCL-XL reversed the suppression in cell survival and apoptosis (Fig. 3e, f), suggesting that miR-876 mediates its tumor suppressor role largely due to the BLC-XL inhibition.

### Stably overexpressing miR-876 suppresses in vivo tumor growth and induces apoptosis

**Fig. 4:**
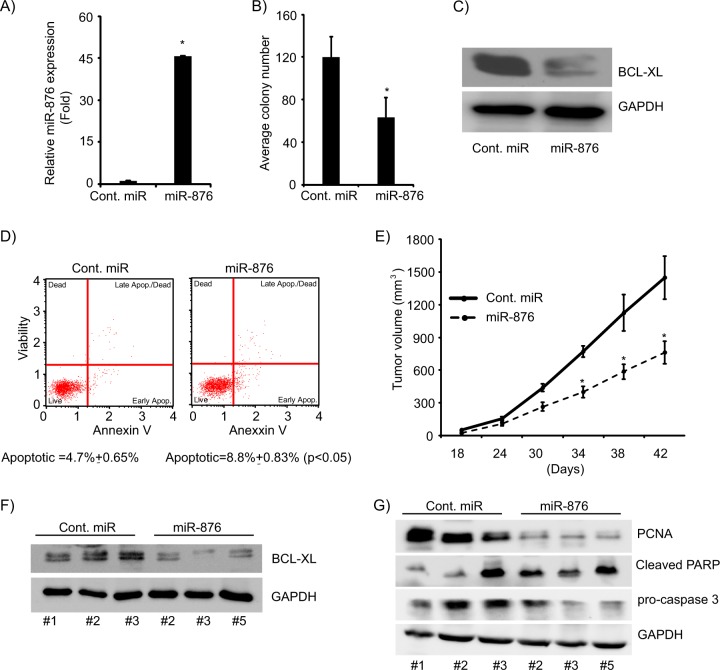
Stable overexpression of miR-876 suppresses in vivo tumor growth. **a** Stable overexpression of miR-876 in the KMCH cell line. **b** Colony formation ability of KMCH cells stably expressing miR-876. **c** BCL-XL protein expression levels in KMCH cells expressing miR-876. **d** Stable expression of miR-876 induces apoptosis. **e** miR-876 expression suppresses in vivo tumor cell growth. **f** miR-876 suppresses BCL-XL expression in tumor in vivo. **g** Western blot analysis showing expression of PARP, caspase 3, and PCNA in miR-876-expressing in vivo tumor samples. *p < 0.05

Next, we investigated the effects of miR-876 stably expressing in CCA cell lines. miR-876 expressing vector was transfected into KMCH cells and stably expressing miR-876 cells were generated. miR-876 expression was confirmed by employing miR-qRT-PCR (Fig. 4a). KMCH cells expressing miR-876 exhibited substantial suppression in ability to form colonies (Fig. 4b). BCL-XL expression was significantly suppressed in KMCH cells stably expressing miR-876 (Fig. 4c). Stable expression of miR-876 induced apoptosis in KMCH cells (Fig. 4d). Furthermore, the in vivo growth of KMCH cells stably expressing miR-876 was significantly suppressed (Fig. 4e) when compared with tumor cells expressing a control vector. In comparison with control, BCL-XL protein expression was reduced in tumors derived from KMCH cells stably expressing miR-876 (Fig. 4f). Expression of the proliferative marker, PCNA, was reduced in miR-876-expressing tumors, whereas, cleaved-PARP expression was elevated in miR-876 expressing tumors. Caspase 3, another apoptotic marker, exhibited significant cleavage in miR-876-expressing tumors (Fig. 4g) indicating apoptotic induction and activation.

### Effects of miR-876 expression in a patient-derived xenograft (PDX)

**Fig. 5:**
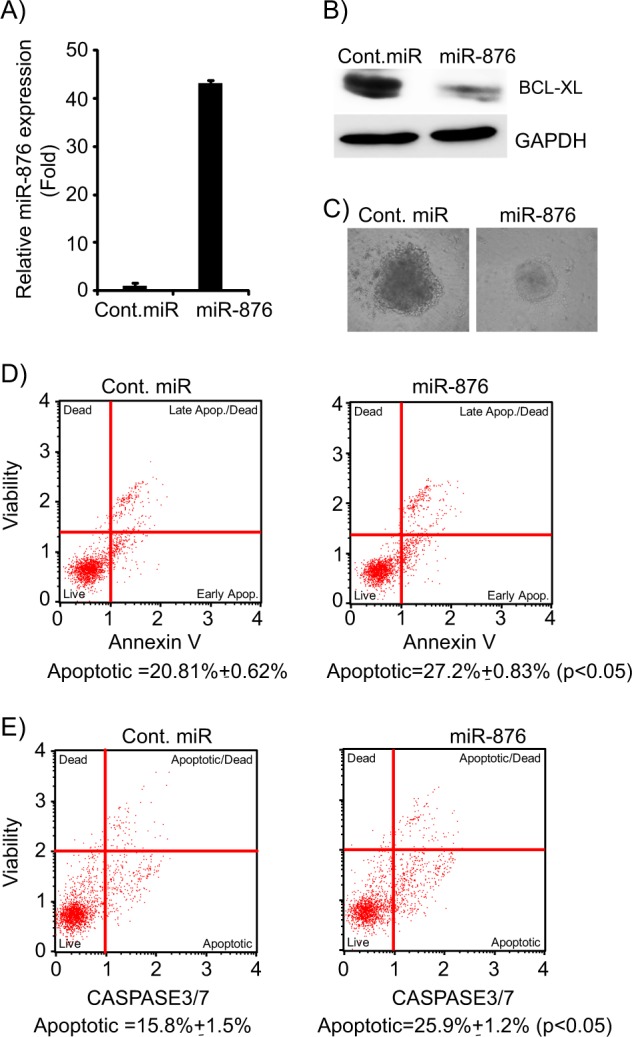
miR-876 overexpression regulates proliferation and apoptosis in a patient-derived xenograft cell line. **a** miR-876 overexpression in the CHNG6 cell line. **b** BCL-XL expression levels folllowing miR-876 overexpression in CHNG6 cells. **c** miR-876 overexpression suppresses cellular spheroid formation. **d** miR-876 overexpression induces apoptosis in CHNG6 cells. **e** miR-876 induces caspase 3/7 activity in CHNG6 cells

A patient-derived xenograft (PDX) mouse model (CHNG6) was successfully generated in NOD/SCID mice from a patient tumor sample. CHNG6 cells were grown in culture as spheroids under tumor stem cell conditions, without fetal bovine serum, to better conserve the phenotype and genotype of the original tumor, and to mimic a 3D tumor tissue environment^11^. miR-876 overexpression (Fig. 5a) substantially suppressed expression of its functional target (i.e., BCL-XL) in CHNG6 cells (Fig. 5b). miR-876 overexpression in CHNG6 cells significantly reduced the area of cellular spheroid formation indicating a suppressive role of miR-876 on tumor cell proliferation (Fig. 5c). CHNG6 cells expressing miR-876 had elevated apoptosis index in comparison with cont. miR (Fig. 5d). Furthermore, a substantial increase in caspase 3/7 expression was observed in CHNG6 cells expressing miR-876 (Fig. 5e). These findings validate the tumor suppressor role of miR-876 not only in established CCA cell lines but also in a novel CCA patient-derived xenograft derived cell culture, which more closely resembles the patient’s original tumor.

## Discussion

CCA, a neoplasm of epithelial cells lining bile ducts, can originate in any portion of the biliary tree^1^. CCA is a rare cancer and its incidence rate is 8% in the US^12^. However, the incidence of CCA is increasing globally^13,14^ and most of them arise without any known predisposition^15^. Thus, there is an unmet clinical need to investigate and identify new novel biomarkers of CCA progression, as well as safe and effective treatments. Dysregulation of miRNAs, along with functional or biomarker role, has been increasingly demonstrated in different tumor types. However, in CCA, the role of miRNAs has not been fully elucidated.

Here, we investigated the biological and functional significance of miR-876 in CCA. miR-876 is not a widely studied miRNA, with recently described roles in atherosclerosis^16^ and lung cancer^17^. Our analysis indicated that miR-876 copy number was significantly suppressed in CCA tumor samples, accompanied by downregulation of its expression in CCA tumor specimens and cell lines. The promoter region of miR-876 contains a methylated CpG island, representing a possible mechanism for its suppressed expression, although additional studies are required to confirm this hypothesis. We demonstrated that BCL-XL, an antiapoptotic gene with a key role in tumor progression, is a direct functional target of miR-876. BCL-XL protein levels were significantly suppressed by miR-876 overexpression suggesting a posttranslational modulation of BCL-XL. miRNAs induce translational repression of their targets by interacting with their 3′UTR. They have been reported to target other regions, including the 5′UTR, the coding sequence, and promoter sequences. miR-876 expression was suppressed and expression levels of BCL-XL, substantially upregulated in CCA tumor samples and cell lines when compared with normal controls, suggest that BCL-XL overexpression may be in part due to the loss of miR-876 expression. BCL-XL is a pro-proliferative gene, and its overexpression in CCA samples may impart a proliferative advantage. To affirm that miR-876 regulates BCL-XL and reduces tumor cell growth, we overexpressed BCL-XL in CCA cells and it reversed the suppression of cell growth and apoptosis caused by miR-876 overexpression. These findings suggest that miR-876 regulates CCA cell proliferation and apoptosis by regulating BCL-XL.

BCL-XL, a BCL2 family member, is widely expressed in many tumors including hepatocellular cancer (HCC), pancreatic cancer, and lung cancer^18–20^. BCL-XL is upregulated in one-third of HCCs^21^, mediates an antiapoptotic role^22^, and has a prognostic implification in HCC^23^. BCL-XL, along with other antiapoptotic BCL2 family members, plays an important role in resisting radiotherapy or chemotherapy induced apoptosis^24^. BCL-XL also plays a critical role in autophagy and apoptosis^25^. BCL-XL overexpression fosters chemotherapeutic drug resistance^26,27^ and also inhibits apoptosis induced by several stress-inducing agents^21,28^. Therefore, targeting BCL-XL is an attractive methodology in cancer to subdue the inherent resistance to apoptosis. The recent development of BCL2 family inhibitors mimicking the BH3 domain has emerged as a rationale strategy to target this family of antiapoptotic genes in human cancer^29^. Employing miRNAs to target BCL-XL represents an alternative approach to target it, along with other BCL2 family members, to suppress their antiapoptotic role. Our study has demonstrated the functional significance of miR-876 in negatively regulating BCL-XL expression and in turn suppressing cellular growth and inducing apoptosis of CCA cells. The reduction of in vivo tumor growth following miR-876 overexpression was accompanied by induction of the apoptotic markers PARP and caspase 3/7, indicating the ability of miR-876 to activate the apoptotic cascade and inhibit CCA cell proliferation. Although further studies need to be conducted to elucidate the functional role of miR-876 in CCA, this study demonstrates a key role for miR-876, in part by suppressing cellular proliferation and inducing apoptosis. Our results were supported by use of a PDX model to corroborate the functional significance of miR-876. As PDXs more closely resemble and mimic the original tumor, this provides additional evidence that miR-876 may regulate expression of BCL-XL in CCA patients.

In conclusion, we have demonstrated that expression of miR-876 is suppressed in CCA cell lines and tissues, whereas, BCL-XL expression is elevated. Overexpression of miR-876 suppressed BCL-XL expression, proliferative ability of CCA cells, and induced apoptosis. Taken together, these findings describe a promising and novel therapeutic role for miR-876 in CCA.

## Materials and methods

### Cell culture and vectors

The CCA HuCCT1 and HuH28 were obtained from the Japanese Collection of Research Bioresources Cell Bank (JCRB, Japan), KMCH, KMBC, and H69 were a kind gift from Dr Gregory Gores, Mayo Clinic, MN. Normal human cholangiocyte line (H69) was cultured as described earlier^30^. The CCA cell lines HuCCT1, KMCH, KMBC, and HuH28 were grown in RPMI (Thermofisher Scientific, South San Francisco, CA) with 5% fetal bovine serum (JR Scientific, Woodland, CA) 1x penicillin/streptomycin in an incubator at 37 °C temperature and 5% CO_2_. Mycoplasma contamination tests were negative for all cell lines. Plasmids pCMV6-BCL-XL and pCMV6-Entry were obtained from Origene (Origene Technologies, Rockville MD). The dual luciferase pmirGLO vector (Promega, Madison, WI), pEZX-MR04-miR-876, and pEZX-MR04 control vectors (GeneCopoeia, Rockville MD) were purchased. Pre-miR-miRNA precursor molecule-negative control (referred now as cont. miR) and pre-miR-miRNA-876 precursor (referred to as miR-876) and their corresponding miRNA Taqman probes were purchased from Thermofisher Scientific. Lipofectamine-2000 (Invitrogen Life Technologies, Carlsbad, CA) was employed for transient transfection as per the manufacturer’s instructions. In brief, 50 nM of cont. miR or miR-876 were used for transfection. miRNAs were mixed with lipofectamine in serum-free medium and the reaction mixture was added to CCA cells for 4 h, after which the media was aspirated and replaced.

### Extraction of RNA and miRNA

RNA extraction was performed employing RNeasy Mini Kit (Qiagen, Valencia, CA) as per the manufacturer’s instructions, whereas, miRNA extraction from cell lines and nine human tumor tissues was performed by utilizing the mirVana miRNA extraction kit (Thermofisher Scientific) according to the manufacturer’s instructions.

### Quantitative real-time PCR

TaqMan MicroRNA Assays and Gene Expression Assays were used to analyze mature miRNA and mRNAs, respectively, according to the manufacturer’s protocol (Thermofisher Scientific). Nanodrop (Thermofischer Scientific) was used to determine the RNA concentrations. All RT reactions were performed in a 7500 Fast RT-PCR System (Thermofisher Scientific). RNU44 or HPRT (Thermofisher Scientific) were used as endogenous controls to normalize the expression as indicated. Quantification of relative expression was performed utilizing 7500 Fast RT Sequence detection system software (Thermofisher Scientific). Three technical replicates were run for each sample and for no-template controls. Comparative Ct method was employed to determine the relative expression. Results are based on mean of biological replicates, three for cell lines and two for tissue samples.

### Colony formation assay

To determine the ability of cells to generate colonies, 500–700 cells were cultured in a six-well plate till visible colonies were observed. Crystal violet was used to stain the colonies. Plates were air-dried and colonies were counted. Experiments were repeated three times in triplicates.

### Annexin and caspase 3/7 assays

These assays were carried out by using the Muse Annexin V apoptosis kit, and Muse Caspase 3/7 kit (EMD Millipore, Hayward, CA) following the manufacturer’s instructions. Experiments were repeated twice in triplicates

### Western blot analysis

Protein extraction was performed using RIPA buffer containing dual protease and phosphatase inhibitors (1X Halt protease and phosphatase inhibitor cocktail, Pierce, Rockford, IL). SDS/polyacrylamide gel electrophoresis (PAGE) was performed using 10–25 μg of protein. Specific antibodies against BCL-XL #2764, Cleaved-PARP #5625, Caspase 3 #9622, PCNA #13110 (Cell Signaling Technology, Danvers, MA), and GAPDH (#MAB374 EMD Millipore, Hayward CA) were used. Experiment was repeated three times.

### Luciferase reporter assays

BCL-XL 3′UTR region harboring complementary binding sequence for miR-876 was cloned in the pmirGLO dual-luciferase vector (Promega, Madison, WI), and named as BCL-XL-UTR. A mutated sequence of BCL-XL 3′UTR complementary binding to miR-876 was cloned in the same vector and named as BCL-XL-Mut. Cells were transiently transfected with wild-type or mutant luciferase reporter vectors and miR-876. Dual Luciferase Assay (Promega, Madison, WI) was performed to measure the firefly luciferase activities 24 h post transfection. Renilla luciferase activity was used as normalizing control. Experiments were repeated three times in triplicates.

### Generation of stable cells and in vivo study

KMCH cells were transfected with pEZX-MR04 control and pEZX-MR04–876 vectors and selected with puromycin (1 µg/mL). Stable transformants were sorted based on GFP expression using FACS Aria II (BD Biosciences). A total of 1 × 10^6^ cells (KMCH-pEZX-MR04 or KMCH-MR04–876) were injected into nu/nu mice (The Jackson Laboratory) subcutaneously (*n* = 6) for each condition as described previously^31^. The number of mice utilized for this experiment was adequate to achieve statistical power. Animals were not randomized as both groups are distinct from each other and the investigator was not blinded. Tumors were measured using caliper, and tumor volumes were determined by the formula (length × width × width)/2. The CPMC Research Institute guidelines were followed for animal care.

### Patient-derived xenograft mouse model

The acquisition of tumor tissue was performed in accordance with the Institutional Review Board protocol approved at California Pacific Medical Center. Informed consent was obtained from the patient (Male, 58 years old, tumor moderately differentiated, and stage IV) in accordance with approved institutional guidelines. Fresh tumor tissue was placed in a 50 mL falcon tube containing Hypothermosol FRS media (BioLife Solutions, Bothell, WA) and transported on wet ice. Tissues were processed for implantation within 1–2 h of resection. Implantation of a tumor fragment (3 × 3 mm) was performed into the flank of NOD-SCID (NSG) mice (The Jackson Laboratory). Mice were regularly monitored and once a successful xenograft developed, mice were euthanized and tumors harvested to generate PDX cell lines. Cells generated from the PDX (referred to as CHNG6), were validated by short tandem repeat DNA profiling by ATCC. The profile of CHNG6 was confirmed human as it did not match with any ATCC database or Deutsche Sammlung von Mikroorganismen und Zellkulturen GmbH DSMZ database cell line. CHNG6 were grown as spheroids in DMEM-F12 media without fetal bovine serum, 1xB27 (Thermofisher Scientific), 20 ng/mL EGF, 20 ng/mL FGF (Peprotech, Rocky Hill, NJ), heparin, and 1× penicillin/streptomycin (Thermofisher Scientific) in an incubator at 37 °C temperature and 5% CO_2_.

### Statistical analysis

For statistical analysis, the Student’s *t*-test and two-tailed *p* values < 0.05 were considered statistically significant. Data are represented through mean with standard bars represent standard deviation. Samples were not excluded from any analysis and no statistical method was used to predetermine sample size.

## Supplementary information

Supplemental Figure 1

Supplemental Figure 2

Supplemental Figure 3

Supplemental Figure Legends
