# Supplementary material for: Novel tumor suppressor role of miRNA-876 in cholangiocarcinoma
Source: Oncogenesis. 2019 Aug 13;8(8):42. doi: 10.1038/s41389-019-0153-z (PMC6692334; doi:10.1038/s41389-019-0153-z)
Supplement: Supplementary file 4 — Supplemental Figure Legends [file 41389_2019_153_MOESM4_ESM.docx]

**Supplemental Figure Legends**

**Figure 1: miR-876 has no effect on BCL-XL mRNA expression.**

A-B) Relative BCL-XL mRNA expression levels in KMCH and HuCCT1 cells after miR-overexpression.

**Figure 2: Effects of miR-876 overexpression on expression of MCL1 and BCL2.**

Western blot analysis showing expression of MCL1 and BCL2 in KMCH A) and HuCCT1 B) following miR-876 overexpression.

**Figure 3: miR-876 overexpression regulates cellular proliferation and induces apoptosis.**

A) miR-876 overexpression in the HuCCT1 cell line. B) Average colony number of HuCCT1 cells after miR-876 overexpression. C-D) miR-876 induces apoptosis and increases caspase 3/7 activity in HuCCT1 cells.
